# Progestin-Primed Ovarian Stimulation Protocol for Patients in Assisted Reproductive Technology: A Meta-Analysis of Randomized Controlled Trials

**DOI:** 10.3389/fendo.2021.702558

**Published:** 2021-08-31

**Authors:** Shaogen Guan, Yuezhi Feng, Yonghan Huang, Jia Huang

**Affiliations:** ^1^Reproductive Medicine Center, The First People’s Hospital of Foshan, Foshan, China; ^2^Reproductive Medicine Center, The First Affiliated Hospital of Sun Yat-Sen University, Guangzhou, China

**Keywords:** progestin-primed ovarian stimulation, progesterone, assisted reproductive technology, ovarian stimulation, premature LH surge, ovarian hyperstimulation syndrome, pregnancy outcome

## Abstract

**Objectives:**

Progestin-primed ovarian stimulation (PPOS) is a new ovarian stimulation protocol that can block the luteinizing hormone (LH) surge through progesterone instead of traditional down regulating or gonadotropin-releasing hormone (GnRH) antagonist, and in order to achieve multi-follicle recruitment. This paper aims to investigate the effectiveness of PPOS and its suitability for infertile patients with different ovarian reserve functions.

**Methods:**

We searched published randomized controlled trials (RCTs) about PPOS on Cochrane Library, PubMed, Embase, and Web of Science. The search period spanned from January 1, 2015 to November 16, 2020. The data were extracted, and the meta-analysis was performed on ovarian stimulation as well as embryological and clinical outcomes. The outcomes were pooled by a random effects model, and the risk of heterogeneity was evaluated. Subgroup analysis was performed for different ovarian reserve patients.

**Results:**

The clinical pregnancy rates and live birth or ongoing pregnancy rates with the PPOS protocol were not different from those with the control group. In the diminished ovarian reserve (DOR) subgroup, the PPOS protocol had a lower rate of premature LH surge [RR = 0.03, 95% CI = 0.01 to 0.13, *p* < 0.001]. The PPOS protocol had a lower rate of ovarian hyperstimulation syndrome (OHSS) [RR = 0.52, 95% CI = 0.36 to 0.76, *p* < 0.001, *I* ^2^ = 0.00%]. The secondary outcomes showed that the number of oocytes retrieved, MII oocytes, and viable embryos was higher than that of the control protocol in DOR patients [(MD = 0.33, 95% CI = 0.30 to 0.36, *p* < 0.001), (MD = 0.30, 95% CI = 0.27 to 0.33, *p* < 0.001), (MD = 0.21, 95% CI = 0.18 to 0.24, *p* < 0.001)] and normal ovarian reserve (NOR) patients [(MD = 1.41, 95% CI = 0.03 to 2.78, *p* < 0.001), (MD = 1.19, 95% CI = 0.04 to 2.35, *p* < 0.001), (MD = 1.01, 95% CI = 0.21 to 1.81, *p* = 0.01)].

**Conclusion:**

The findings suggest that PPOS is an effective ovarian stimulation protocol and is beneficial for patients with different ovarian reserve functions, which needs to be validated in more RCTs with larger samples.

## 1 Introduction

The controlled ovarian stimulation (COS) protocol is a major step in assisted reproductive technology (ART) (1). For greater COS efficacy, the premature luteinizing hormone (LH) surge (2, 3), which is caused by increasing plasma estradiol produced by multiple growing follicles, should primarily be prevented. Failing to control the LH surge prior to the scheduled time will lead to spontaneous ovulation (4), decreased oocyte yield, or premature progesterone elevation causing endometrial-embryo asynchrony (5–7). For several years, the conventional COS protocol was commonly associated with gonadotropin releasing hormone (GnRH) analogues to prevent a premature LH surge (8, 9). Despite their overall effectiveness, the LH surge occurs in 3-10% of all *in vitro* fertilization (IVF) cycles (10). Furthermore, the utilization of GnRH analogues is burdened by high costs and poor adherence to the daily subcutaneous administration. In recent years, GnRH antagonists are favored over GnRH agonists owing to the advantages of the short duration of injection time and the reduced risk of ovarian hyperstimulation syndrome (OHSS) (11, 12). However, the GnRH antagonists are still expensive and require daily injection.

Over the past few years, the research interest has focused on the replacement of GnRH analogues by progestins for controlling the LH surge due to the adverse attributes of the GnRH analogues. Progestin was thought to be an alternative agent for the suppression of premature LH surge during COS. Endogenous progesterone could hinder the rise of LH in the event that no spontaneous LH surge occurred during COS in the luteal phase in certain studies (13–15). Progesterone reduces GnRH’s pulsatility from the hypothalamus, thus inhibiting the LH release associated with increased estradiol levels. Therefore, a new strategy for COS, i.e., PPOS, was gradually investigated. In 2015, Kuang (13) first used Medroxyprogesterone acetate (MPA) for LH suppression in COS, which resulted in similar outcomes with short agonist protocol. Subsequent studies also demonstrated the efficacy of progesterone in preventing LH elevation during ovarian stimulation. In contrast to GnRH analogues, the use of progestin for LH suppression is associated with the promising advantages of oral administration, user convenience, and low cost (14, 15). Concomitantly, however, the endometrium is not allowed for fresh embryo transfer because early exposure to the progesterone would result in endometrial advancement (16). In order to overcome the adverse effect of the progesterone on endometrium, one strategy is to freeze all the embryos and defer the embryo-transfer in a future frozen-thawed replacement cycle (FET). This was enabled by the development of advanced cryopreservation techniques.

Thanks to the economic and clinical convenience, the PPOS protocol has gained considerable popularity nowadays. Several investigations about the use of PPOS protocol in different ovarian reserve patients had been reported. Nevertheless, information about the effectiveness of progestins compared to GnRH analogues in various populations of patients is limited; for instance, information pertaining to whether PPOS has the same effect or is safer than the conventional COS protocols in all patient populations is limited. The purpose of this systematic review was to investigate whether PPOS has the same results of LH suppression in COS and achieves similar pregnancy outcomes with conventional protocols. This study will hopefully provide statistical evidence for clinicians on PPOS use in the treatment of infertility.

## 2 Methodology

### 2.1 Data Sources and Search Strategy

We searched the Cochrane Library, PubMed, Embase, and Web of Science. The search period spanned from January 1, 2015 to November 16, 2020, and as a result, PPOS was first proposed by Kuang Yanping in 2015 (13). At the same time, we manually searched previously published meta-analyses and references to the studies we included. Only studies published in English as a full-text article were included. The search strategy is available in **Supplementary Appendix 1**. The protocol for the present systematic review was registered in Prospero (CRD42021232908).

### 2.2 Study Selection

Two authors (SG, YF) independently selected the studies from each of the eligible study, and all the selected studies were confirmed by a third author (JH). The inclusion criteria was randomized controlled trials (RCTs) of infertility patients undergoing ART in one COS cycle. The intervention for IVF was PPOS, and the control interventions included the GnRH antagonist protocol, the GnRH agonist protocol, as well as the natural cycle. The primary outcome was the incidence of premature LH surge and OHSS, the clinical pregnancy rate per woman, and the live birth or ongoing pregnancy rate per woman. The secondary outcomes were as follows: number of oocytes retrieved, number of metaphase two (MII) oocytes, number of viable embryos, duration of gonadotropin (Gn) treatment, amount of Gn administered, and miscarriage rate per woman. We excluded the following studies: (1) patients with endometriosis or cancer; (2) unpublished articles and conferences; and (3) duplication of data (i.e., from different authors of the same study) and incomplete or missing data.

### 2.3 Data Extraction and Quality Assessment

Two investigators (SG, YF) independently extracted data and assessed the quality of the selected studies according to the Cochrane Collaboration’s tool for randomized controlled trials (17). Items will be evaluated in three categories: low risk of bias, unclear bias, and high risk of bias. The following characteristics will be evaluated: random sequence generation (selection bias), allocation concealment (selection bias), blinding of participants and personnel (performance bias), incomplete outcome data (attrition bias), selective reporting (reporting bias), and other biases. The results from these questions will be graphed and assessed using Review Manager 5.3.

### 2.4 Data Synthesis and Data Analysis

#### Statistical Analysis

We used the SATA version 16 for data analysis. For dichotomous outcome measures, risk ratio (RR) and its 95% confidence intervals (CIs) are presented. For continuous outcome measures, the mean difference and its 95% CIs are presented. A random effects model was employed based on the heterogeneity of the data as assessed by the *I* ^2^ statistic that reflects the proportion of the observed dispersion between studies (18).

We performed subgroup analysis for different ovarian reserve patients (including DOR, NOR, and PCOS subgroups) and different treatment protocols. We did not analyze publication bias because the number of included literatures was less than 10.

## 3 Results

### 3.1 Search Results

**Figure 1:**
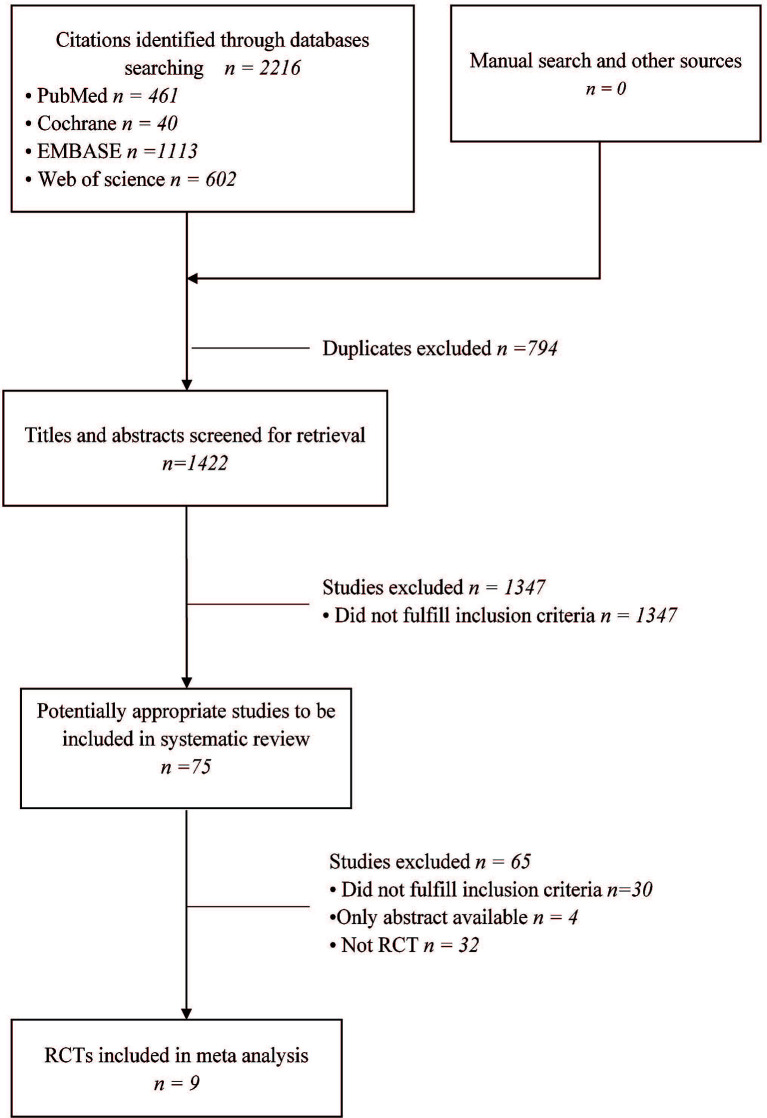
PRISMA flow diagram of study selection for the systematic review and meta-analysis.

A preliminary search was conducted to obtain 2,216 relevant studies. After reading the titles, abstracts, and full texts of the studies and eliminating duplicate studies as well as those that did not meet the inclusion criteria, the authors retained nine studies which finally met the inclusion criteria. A total of 1,885 cycles were integrated in the nine studies, including 942 cycles for PPOS and 943 cycles for the control group (**Figure 1**).

### 3.2 Study Characteristics and Bias Assessment

All research were randomized controlled studies. Among them, two studies concerned diminished ovarian reserve patients, five studies concerned normal ovarian reserve patients, and two studies related to PCOS patients. In the control group, five studies were antagonist protocols, one study was a natural cycle, two studies were short protocols, and one study was a GnRH-a long protocol. In addition, seven studies used HMG and two studies used FSH for ovulation induction. The basic information of the included studies is shown in **Table 1**.

**Table 1:** Characteristics of included studies.

Study	Country	Ovarian reserve	PPOS Group			Control Group			Gn	Outcome
			Sample Size	Age (year)	Progesterone	Sample Size	Age (year)	Intervention		
Chen et al. (19)	China	DOR	102	37.3 ± 4.7	MPA 10mg qd	102	37.8 ± 4.7	Natural Cycle	HMG	①②③④⑥⑦⑧⑨⑩
Chen et al. (20)	China	DOR	170	34.8 ± 4.2	MPA 10mg qd	170	35.1 ± 4.1	Antagonist Protocol	HMG	①②③④⑥⑦⑧⑨⑩
Eftekhar et al. (21)	Iran	PCOS	58	28.5 ± 3.6	Dydrogesterone 20mg qd	60	29.0 ± 3.6	Antagonist Protocol	FSH	①③⑤⑥⑦⑧⑨⑩
Ghasemzadeh et al. (22)	Iran	NOR	50	30.2 ± 6.2	Utrogestan 100 mg bid	50	32.4 ± 6.6	Antagonist Protocol	FSH	②⑥⑧⑨⑩
Hossein Rashidi et al. (23)	Iran	NOR	97	32.7 ± 4.6	Dydrogesterone 20mg qd	95	33.2 ± 5.2	Antagonist Protocol	HMG	①②③⑤⑥⑦⑧⑨⑩
Iwami et al. (24)	Japan	NOR	125	34.8 ± 3.5	Dydrogesterone 20mg qd	126	34.2 ± 3.7	Antagonist Protocol	HMG	①②③④⑤⑥⑦⑧⑨
Kuang et al. (13)	China	NOR	150	31.6 ± 3.6	MPA 10mg qd	150	31.0 ± 3.3	Short Protocol	HMG	①②③④⑤⑥⑦⑧⑨⑩
Wang et al. (14)	China	PCOS	60	30.4 ± 3.1	MPA 10mg qd	60	29.9 ± 3.1	Short Protocol	HMG	①②③④⑤⑥⑦⑧⑨⑩
Xi et al. (25)	China	NOR	130	31.4 ± 4.0	MPA 10mg qd	130	31.6 ± 4.2	GnRHa Long Protocol	HMG	①②③④⑤⑥⑦⑧⑨⑩

① Clinical pregnancy rate; ② premature LH surge; ③ Miscarriage rate; ④ Live birth or Ongoing pregnant rate per woman; ⑤ OHSS; ⑥ Gn duration; ⑦ Gn dose; ⑧ Oocytes retrieved; ⑨ MII oocytes; ⑩ Number of embryos.

**Figure 2:**
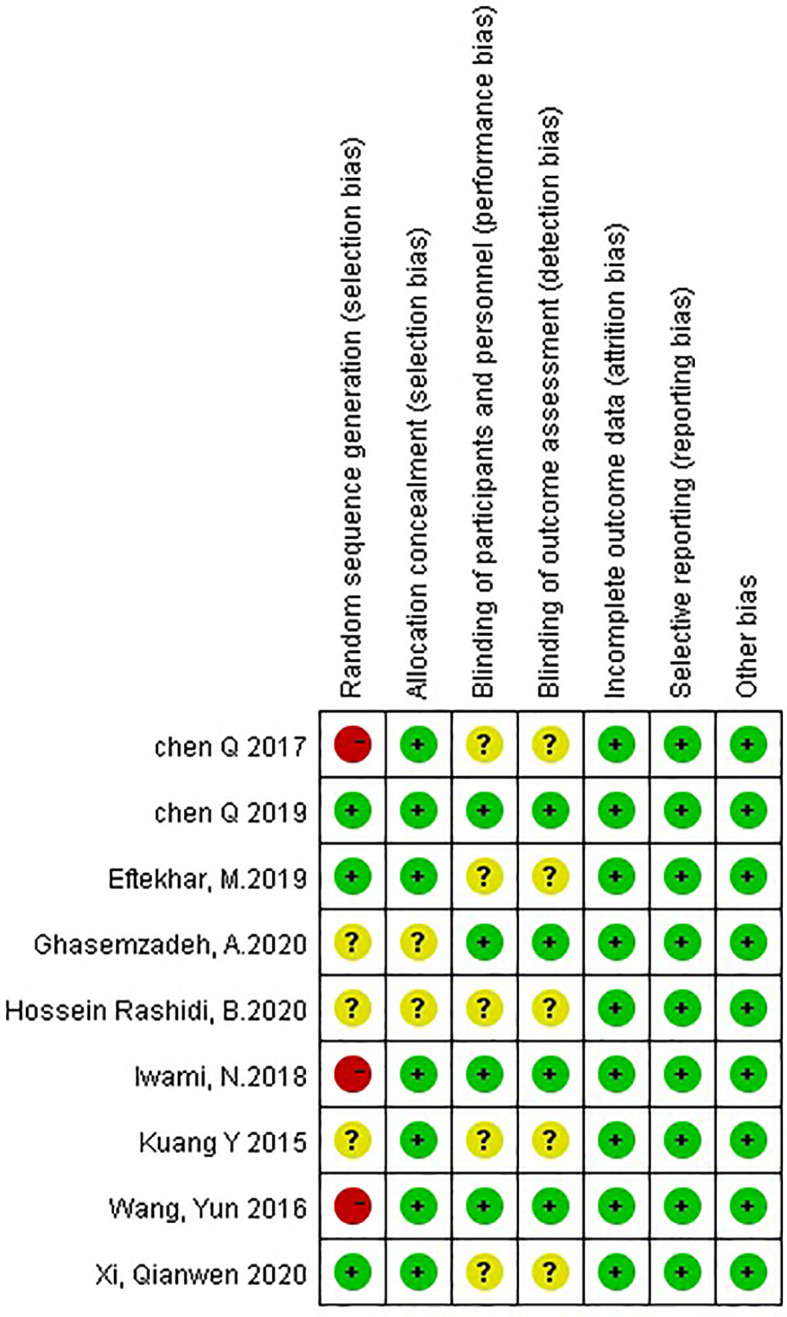
Risk of bias assessment for the randomized studies.

According to the Cochrane Collaboration’s tool for randomized controlled trials, three RCTs adequately generated their randomization sequence, and three were high risk; seven RCTs adequately concealed allocation, and two were unclear risk; four RCTs were low risk for blinding bias, and five others were unclear. All trials were at low risk for attrition bias, reporting bias, and other biases. The full details of the risk of bias assessment for the studies are provided below (**Figure 2**).

## 4 Outcomes

### 4.1 The Primary Outcome

#### 4.1.1 Premature LH Surge

**Figure 3:**
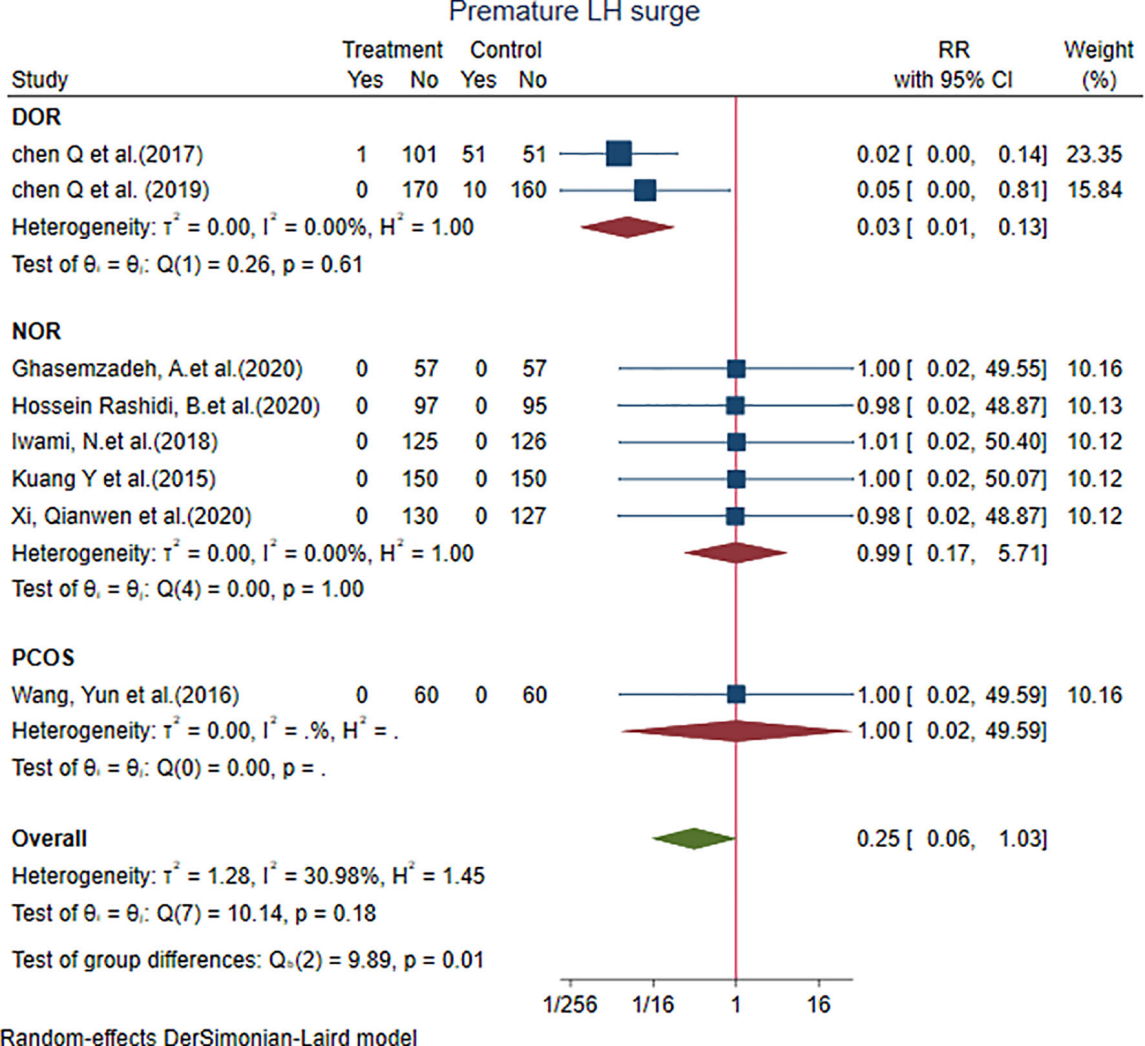
Forest plot of studies of premature LH surge.

Eight studies showed that the premature LH surge with the PPOS protocol was not different from that with the control group [RR = 0.25, 95% CI = 0.06 to 1.03, *p* = 0.05, *I* ^2^ = 30.98%]. We performed subgroup analysis. Two studies in the DOR subgroup [RR = 0.03, 95% CI = 0.01 to 0.13] showed that the PPOS protocol had a lower rate of premature LH surge; the result was statistically significant. Five studies in the NOR subgroup [RR = 0.99, 95% CI = 0.17 to 5.71] and only one study in the PCOS subgroup demonstrated that both subgroups did not show any significant difference with the control group (**Figure 3**).

#### 4.1.2 Clinical Pregnancy Rate per Woman

**Figure 4:**
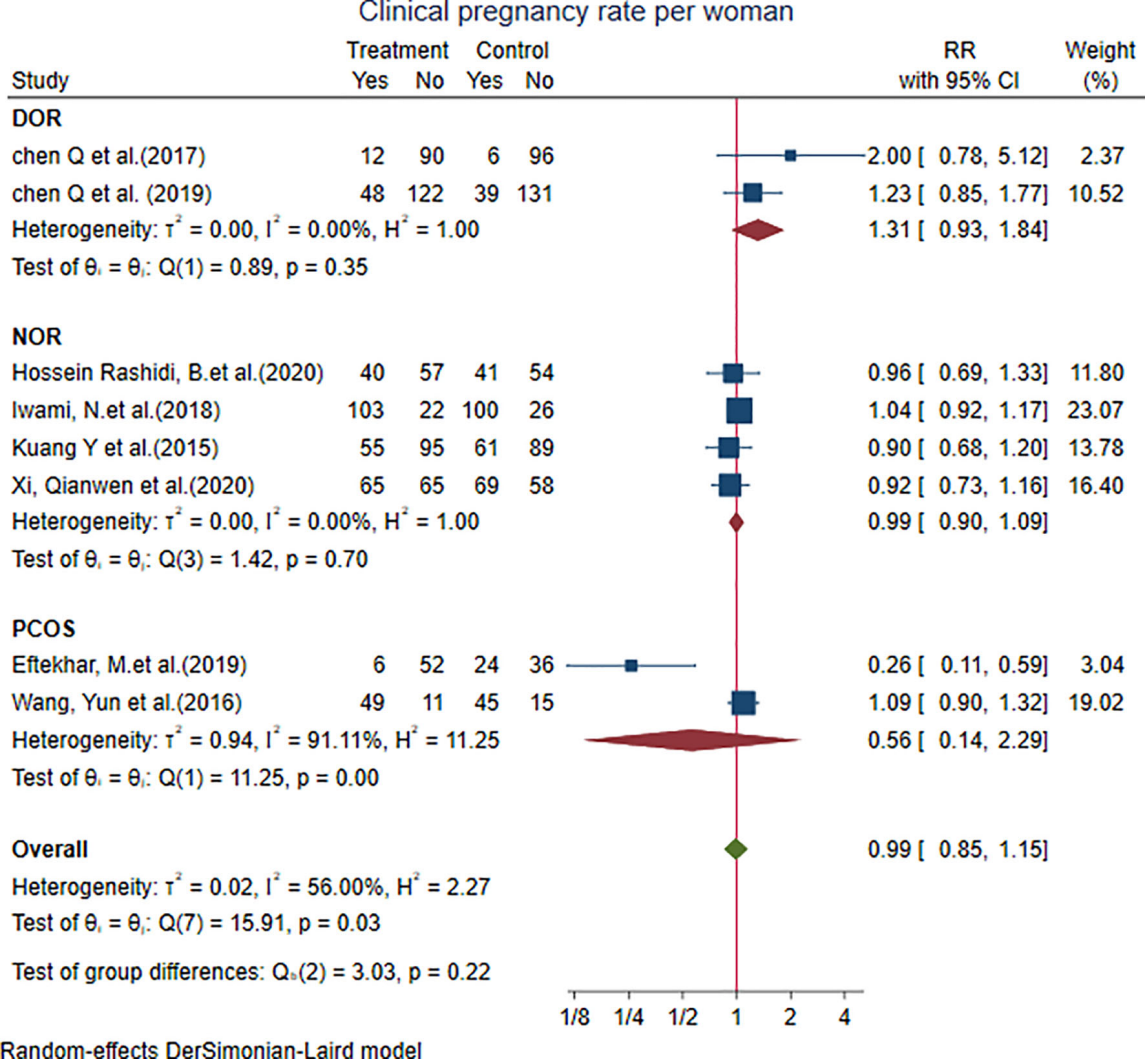
Forest plot of studies of clinical pregnancy rate per woman.

Eight studies showed that the clinical pregnancy rate per woman with the PPOS protocol was not different from that with the control group [RR = 0.99, 95% CI = 0.85 to 1.15, *p* = 0.88, *I* ^2^ = 56.00%]. We performed subgroup analysis: two studies in the DOR subgroup [RR = 1.31, 95% CI = 0.93 to 1.84], four studies in the NOR subgroup [RR = 0.99, 95% CI = 0.90 to 1.09], and two studies in the PCOS subgroup [RR = 0.56, 95% CI = 0.14 to 2.29]. The results did not show any significant difference from the control group (**Figure 4**).

#### 4.1.3 Live Birth or Ongoing Pregnancy Rate per Woman

**Figure 5:**
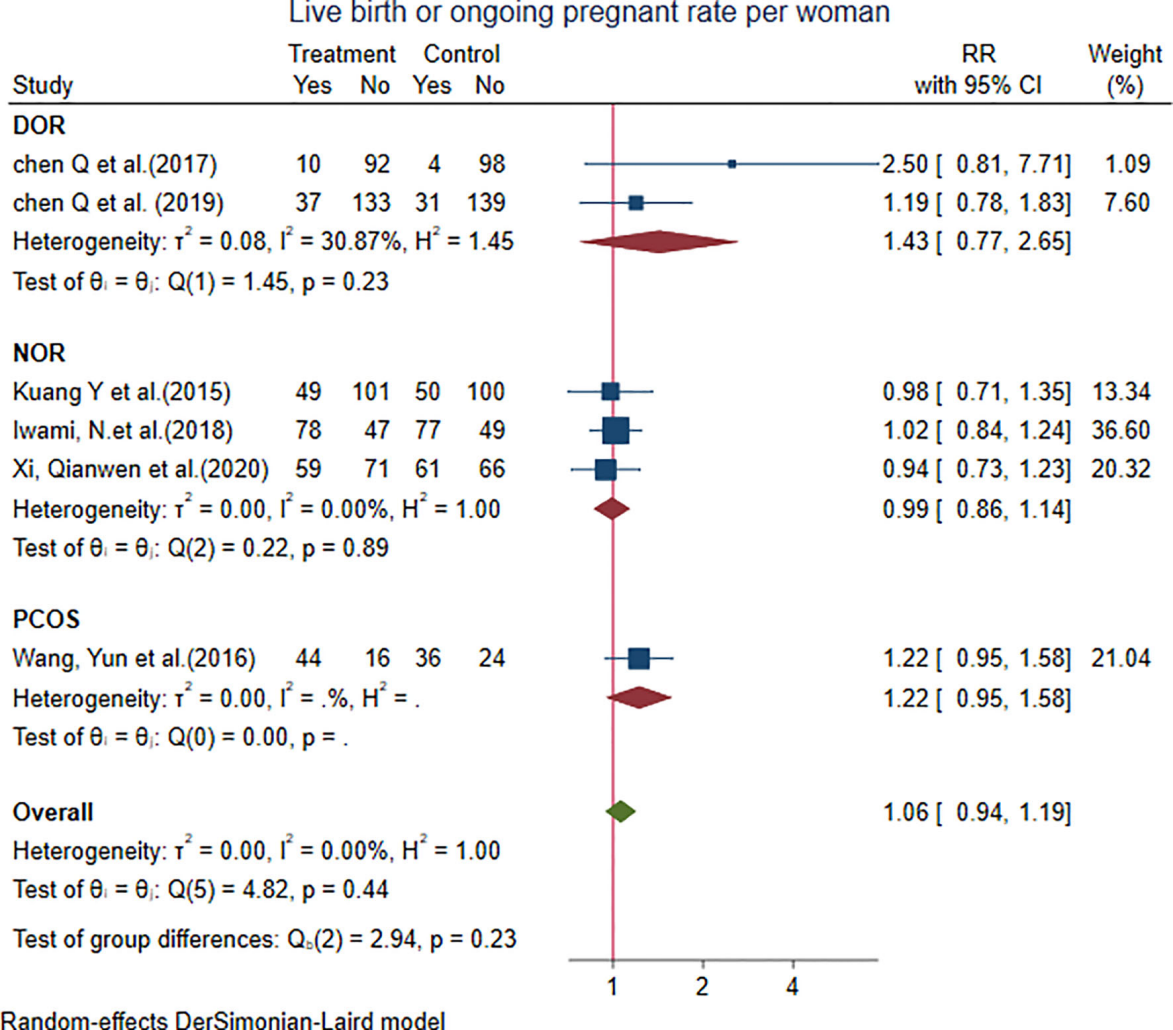
Forest plot of studies of live birth or ongoing pregnancy rate per woman.

Six studies showed that the live birth or ongoing pregnancy rate per woman with the PPOS protocol was not different from that with the control group [RR = 1.06, 95% CI = 0.94 to 1.19, *p* = 0.33, *I* ^2^ = 0.00%]. We performed subgroup analysis: two studies in the DOR subgroup [RR = 1.43, 95% CI = 0.77 to 2.65], three studies in the NOR subgroup [RR = 0.99, 95% CI = 0.86 to 1.14], and only one study in the PCOS subgroup. The results did not show any significant difference from the control group (**Figure 5**).

#### 4.1.4 OHSS

**Figure 6:**
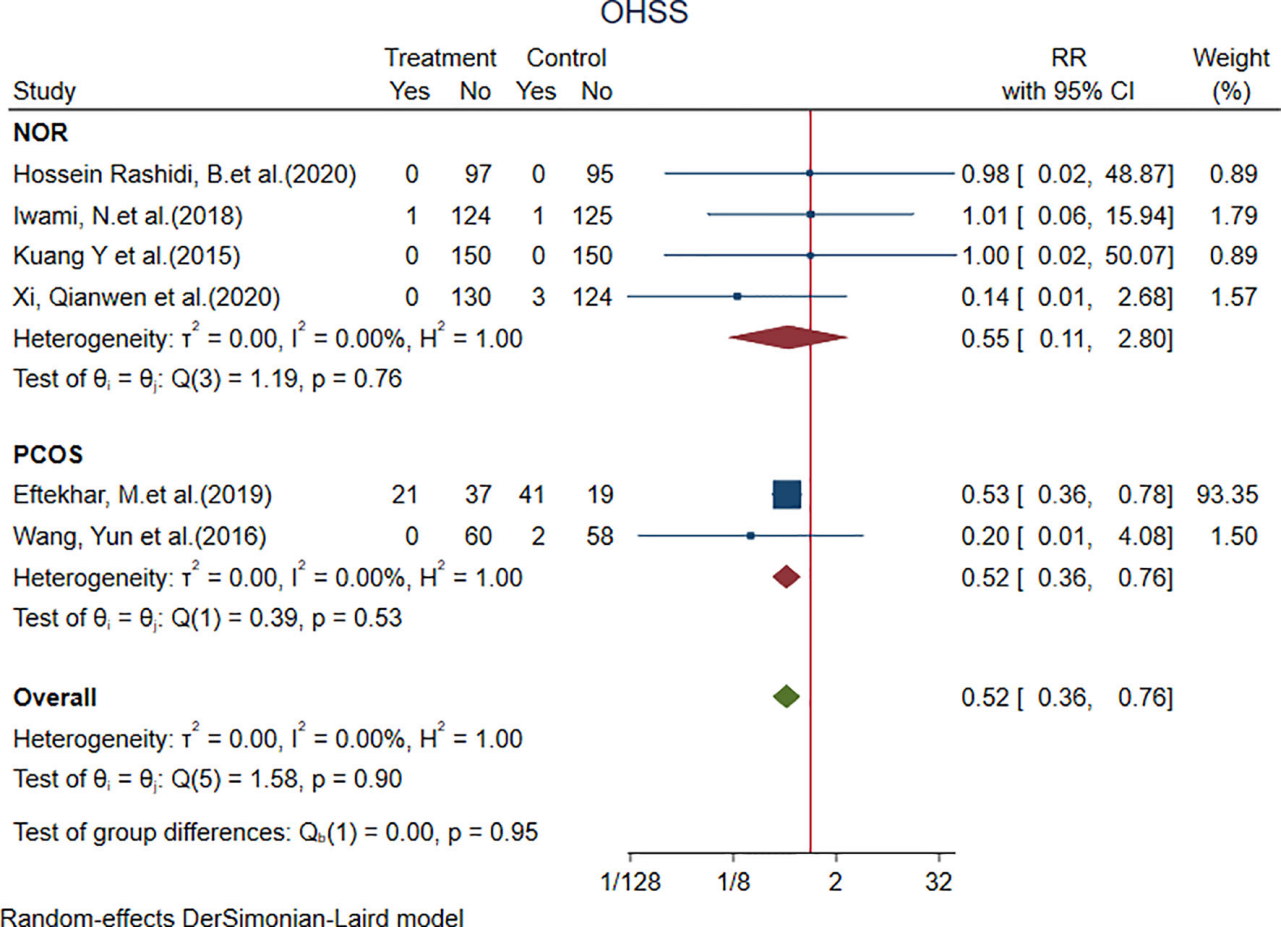
Forest plot of studies of OHSS.

Two studies in the PCOS subgroup [RR = 0.52, 95% CI = 0.36 to 0.76, *p* < 0.001] showed that the incidence of OHSS with the PPOS protocol was different from that with the control group, and the result was statistically significant (**Figure 6**).

#### 4.1.5 Subgroup Analysis for Treatment Protocols

In subgroup analysis for treatment protocols, there was no difference between PPOS and the control group in the clinical pregnancy rate per woman as well as the live birth or ongoing pregnancy rate per woman. One study for natural cycle showed that the premature LH surge with PPOS was significant different from natural cycle, but there was no difference from other treatment protocols. Four studies in antagonist protocol subgroup [RR = 0.54, 95% CI = 0.37 to 0.79, *p* < 0.001] showed that the incidence of OHSS with the PPOS protocol was lower than that with antagonist protocol, but there were no difference in other protocol subgroups (**Supplementary Appendix 3**).

### 4.2 Secondary Outcomes

#### 4.2.1 Gn Duration

Eight studies showed that the Gn duration with the PPOS protocol was not different from that with the control group [MD = -0.15, 95% CI = -1.10 to 0.80 days, *p* = 0.76, *I* ^2^ = 96.08%]. There was no significant difference from the control group in the subgroup analysis.

#### 4.2.2 Gn Dose

Seven studies showed that the Gn dose with the PPOS protocol was not different from that with the control group [MD = 130.46, 95% CI = -205.06 to 465.98 IU, *p* = 0.45, *I* ^2^ = 97.69%]. There was no significant difference from the control group in the subgroup analysis.

#### 4.2.3 LH on Trigger Day

Eight studies showed that the LH on trigger day with the PPOS protocol was not different from that with the control group [MD = -0.24, 95% CI = -1.16 to 0.68, p = 0.61, I2 = 96.58%]. In the subgroup analysis of patients with different ovarian reserve functions, the LH level of DOR patients showed a downward trend in the PPOS group, but there was no statistical difference.

#### 4.2.4 Oocytes Retrieved

Nine studies showed that the number of oocytes retrieved with the PPOS protocol were different from those with the control group [MD = 0.67, 95% CI = 0.04 to 1.30, *p* = 0.04, *I* ^2^ = 72.18%]. In the subgroup analysis, two studies in the DOR subgroup [MD = 0.33, 95% CI = 0.30 to 0.36, *p* < 0.001] and five studies in the NOR subgroup [MD = 1.41, 95% CI = 0.03 to 2.78, *p* < 0.001] showed that the oocytes retrieved in the PPOS protocol were more than in the control group, and the difference was statistically significant. Data from the PCOS subgroup [MD = -1.62, 95% CI = -3.95 to 0.72] showed that the number of oocytes retrieved between the two groups were nearly the same.

#### 4.2.5 MII Oocytes

Nine studies showed that the number of MII oocytes with the PPOS protocol were different from those with the control group [MD = 0.58, 95% CI = 0.01 to 1.15, *p* = 0.04, *I* ^2^ = 72.94%]. In the subgroup analysis, two studies in the DOR subgroup [MD = 0.30, 95% CI = 0.27 to 0.33, *p* < 0.001] and five studies in the NOR subgroup [MD = 1.19, 95% CI = 0.04 to 2.35, *p* < 0.001] showed that the number of MII oocytes in the PPOS protocol were more than that in the control group, and the difference was statistically significant. Data from the PCOS subgroup [MD = -1.84, 95% CI = -4.97 to 1.29] showed that the number of MII oocytes between the two groups were nearly the same.

#### 4.2.6 Viable Embryos

Eight studies showed that the number of viable embryos with the PPOS protocol was not different from that with the control group [MD = 0.36, 95% CI = 0.00 to 0.72, *p* = 0.05, *I* ^2^ = 69.58%]. However, in the subgroup analysis, the DOR subgroup [MD = 0.21, 95% CI = 0.18 to 0.24, *p* < 0.001] and NOR subgroup [MD = 1.01, 95% CI = 0.21 to 1.81, *p* = 0.01] showed that the number of viable embryos in the PPOS protocol was more than that in the control group, and the difference was statistically significant. Two studies in the PCOS subgroup [MD = -0.91, 95% CI = -1.85 to 0.04] showed that the number of viable embryos in the PPOS protocol was not different from that in the control group.

#### 4.2.7 Miscarriage Rate

Eight studies showed that the miscarriage rate with the PPOS protocol was not different from that with the control group [RR = -0.03, 95% CI = -0.35 to 0.29, *p* = 0.85, *I* ^2^ = 0.00%]. There was no significant difference with the control group in the subgroup analysis (**Supplementary Appendix 2**).

## 5 Discussion

In this meta-analysis, PPOS had the same clinical pregnancy rate and ongoing pregnancy rate/live birth rate as the conventional COS protocols in all patient populations. For various populations of patients, PPOS seemed to be favored over the conventional COS protocols owing to certain advantages. In DOR patients, PPOS had been demonstrated to be more efficient in the LH suppression since the rate of premature LH surge was significantly lower in the PPOS protocol. On the other hand, the application of PPOS in NOR patients seemed to have more oocytes retrieved, MII oocytes, and viable embryos. For PCOS patients, PPOS also seemed to be adopted since the incidence of OHSS had been demonstrated to be significantly lower. The findings of this meta-analysis are referenced for clinicians since controversies have always been raised about the application of PPOS in different populations of patients. Our suggestion of an effective impact of progestins for preventing premature LH surge is consistent with that of a previous meta-analysis (26) which included three retrospective studies and two prospective studies but only two RCTs. The evidence of that meta-analysis seemed to be of low quality and the results required confirmation by higher quality studies. Unlike in the previous study, we included nine RCTs that compare progestins with other conventional COS protocols. The nine studies involved a total of 1,885 women, 942 women in the PPOS protocol, and 943 women in the control group. Among the nine RCTs published, eight studies showed that the rate of premature LH surge per woman with the PPOS protocol was not different from the control group. When analyzed by subgroup, two studies compared PPOS with conventional protocol in DOR patients, five studies compared PPOS with conventional protocol in NOR patients, and only one study compared PPOS with conventional protocol in PCOS patients. In DOR patients, progestin had been demonstrated to be more efficient in LH suppression than conventional COS protocols. In the other two subgroups, progestin had the same effect on the prevention of LH surge. In addition, when being analyzed by different COS protocols, the results showed that the incidence of premature LH surge was reduced in the PPOS protocol when it was compared with natural cycle or antagonist protocol. Overall, given the consistency of the results across studies with different participant characteristics and different progestins and studies that have been conducted in various centers, we propose that there is high-quality evidence that supports the effectiveness of progestins in suppressing the LH surge during COS.

A premature LH surge has always been likely to occur in DOR patients who have a small number of antral follicles that grow and mature fast and are prone to premature luteinization (27). Theoretically and clinically, it is more difficult to control LH surge in DOR patients than in those of NOR or PCOS (28). In one of the two RCTs (20) included in our meta-analysis, PPOS had a more robust effect on preventing premature LH surge than GnRH antagonist in DOR patients (0 *vs.* 5.88%, *p* < 0.01). This effect might be due to the fact that GnRH antagonists achieve LH suppression by hindering the competitive GnRH receptors in a direct manner, but the endogenous estrogen-induced GnRH release was still preserved. Antagonists were usually applied on Day 5 of the gonadotropin stimulation, when the leading follicle reached 12-14 mm or when the serum estradiol level reached > 200 pg/ml. As a consequence, premature LH surge may occur before the antagonist application in some cases, especially in advanced age or DOR patients (29–31). On the contrary, progestin inhibits GnRH secretion on the hypothalamus if it is administered during the early part of the cycle before estrogen priming (32–34). Several experiments had shown that higher level of progestin from the early follicular phase is able to inhibit follicular growth and the LH surge by hindering estrogen’s positive feedback on the hypothalamus (35, 36), thus slowing the LH pulse frequency, increasing its amplitude, and decreasing its plasma content (37, 38). In our meta-analysis, however, the LH level on the trigger day in the PPOS protocol was not different from that in the control group. When analyzed by subgroup, the LH level on the trigger day in DOR patients had been demonstrated to be lower in the PPOS protocol than the conventional COS protocol, but the difference was not significant. As a consequence, we assumed that the mechanism underlying progestin on controlling LH may be by an indirect and slow manner, and thus, the serum LH level can be maintained relatively steady and oocyte retrieval can be easily programmed. According to our meta-analysis, progestin has been demonstrated to be superior to GnRH antagonists in controlling premature LH surges in DOR patients. Nonetheless, there was only one RCT included with regard to the comparison with antagonists. Another RCT (19) about the DOR patients compared PPOS with the natural cycle, and the incidence of LH level was reduced by almost 50%. Despite an increasing number of studies demonstrating the effect of progestin, the exact mechanism through which progestin interacts with estrogen to regulate the LH surge is not completely understood.

With regard to the safety of COS, the finding of our meta-analysis is inspiring for the result of a significantly decreased rate of OHSS being observed in patients of PCOS character. PCOS had always been a high risk of OHSS, and the ideal management of PCOS patients would be a protocol that minimizes their OHSS risk while achieving optimal IVF outcomes. One of the RCT by Wang (14) compared PPOS to a short agonist protocol in PCOS patients. None of the patients suffered from the moderate or severe OHSS, while the number of oocytes retrieved and the ongoing pregnancy rate are of no significant difference. However, PPOS in that RCT had used triptorelin 0.1 mg and Human Chorionic Gonadotrophin (HCG) 1000IU, while the short protocol utilized HCG 2000IU for triggering. The amount of HCG is strongly associated with the incidence of OHSS. Therefore, the result should be interpreted with caution. Another RCT (21) had been designed to compare PPOS to the antagonist protocol, the mainstream protocol for PCOS patients. Similar results had been obtained by that RCT, in which cases of mild and moderate OHSS were less in the PPOS group. For better analysis, we also compared the rate of OHSS by different COS protocols and the result had been inspiring for the incidence of OHSS was significantly reduced in the PPOS protocol when it was compared with the antagonist protocol. However, in our meta-analysis, the differences between the number of oocytes retrieved and MII oocytes are not significant in the PCOS, while there are more oocytes retrieved and MII oocytes in the subgroup of DOR or NOR. In DOR patients, decreasing the cycle cancellation rate and increasing the number of oocytes retrieved can augment the chance of transplantation and improve the clinical pregnancy rate. When NOR patients perform PGT, more oocytes retrieved can increase the probability of obtaining normal embryos. Overall, PPOS application in PCOS had been proven to have the advantage of low incidence of OHSS. In line with previous studies (13, 39), progestin prevented the incidence of OHSS in COS at both the follicular and luteal phase. However, the mechanism underlying progestin that prevents OHSS is not clear. Further investigation is needed concerning the role of progestin in controlling OHSS during gonadotropin stimulation.

As mentioned in our meta-analysis, evidence suggests that PPOS provides similar pregnancy rate or ongoing pregnancy/live birth rate compared to conventional protocols. However, a limitation of this meta-analysis is the lack of information about the effect of progestin on oocyte developmental potential and embryo euploidy. Elevated level of progesterone of the trigger day has been verified to decrease cumulative pregnancy rate in IVF cycles, though this adverse impact is mainly for the bad effect of progesterone on endometrium (40). As advanced cryopreservation techniques develop and “freeze all” strategies are accepted widely, the detrimental effect of progestin on endometrial can be avoided by deferring ET. Concerns about long-time exposure to progestin on oocytes and embryos have been raised by clinicians. Until now, relatively few studies have investigated this aspect. A prospective non-inferiority age-matched case-control study (41) which performed preimplantation genetic testing (PGT) had found a substantial similarity of euploid rate of embryos and euploid blastocysts. Euploid rate can be assumed as an important marker for live birth rate and neonatal outcomes. A retrospective study by Huang (42) in 2019 reported no different neonatal outcomes, such as preterm birth, low live birth rate, and major congenital malformations, when comparing PPOS to a short antagonist protocol. Similar reassuring evidences have recently been provided by a meta-analysis (43), with the conclusion that no important harm had been observed with PPOS in terms of congenital malformations. However, the four studies included in that meta-analysis were retrospective cohort studies and lacked randomization. Thus, these studies can be considered to provide low-quality evidence in that regard. Uncertainty about the impact of PPOS on oocytes and embryos still exists. Thus, further well-designed studies are required to achieve confirmation.

Based on the results of our meta-analysis, PPOS provides an appealing management for patients of different characters. With similar pregnancy outcome, PPOS seems to be superior to convention protocols owing to certain advantages, including oral medication, better management of LH surge during COS in diminished ovarian patients, and lower risk of OHSS in PCOS. With regard to concerning costs and the course of treatment, PPOS does not seem to be cost-effective compared to conventional fresh embryo transfer protocols because the application of progestin requires a freeze-all strategy of embryos (44). However, PPOS may be cost-effective when the freeze-all strategy is planned such as in preimplantation genetic testing or fertility-preservation cycles where a GnRH antagonist protocol would otherwise be used.

## Limitations

This study should be interpreted in light of certain limitations. First, this is a study-level meta-analysis that provides average patient characters. The lack of treatment-level data prevents us from assessing the impact of different stimulation protocols on treatment effects. In addition, in order to provide a high-quality meta-analysis, only RCTs had been included in our study, and although a comprehensive search has been conducted, the limited number of studies in each subgroup may reduce the power of detecting smaller significant differences. The criterion of patient characters classification is not completely identical, thus providing bias results.

As mentioned above, a high-quality meta-analysis including more well-designed RCTs about the comparison of PPOS to conventional protocols in patients of different characters is required to increase the strength of our hypothesis-generating findings.

## Conclusion

This meta-analysis indicates that PPOS is an effective ovarian stimulation protocol, which is beneficial for patients with different ovarian reserve functions, and it needs to be validated in more well-designed RCTs with larger samples.

## Data Availability Statement

The original contributions presented in the study are included in the article/**Supplementary Material**. Further inquiries can be directed to the corresponding author.

## Author Contributions

SG and JH conceived the study. SG, YF, YH, and JH participated in the statistical analysis. SG and YF wrote the manuscript. All authors contributed to the article and approved the submitted version.

## Conflict of Interest

The authors declare that the research was conducted in the absence of any commercial or financial relationships that could be construed as a potential conflict of interest.

## Publisher’s Note

All claims expressed in this article are solely those of the authors and do not necessarily represent those of their affiliated organizations, or those of the publisher, the editors and the reviewers. Any product that may be evaluated in this article, or claim that may be made by its manufacturer, is not guaranteed or endorsed by the publisher.
