# Supplementary material for: Progestin-Primed Ovarian Stimulation Protocol for Patients in Assisted Reproductive Technology: A Meta-Analysis of Randomized Controlled Trials
Source: Front Endocrinol (Lausanne). 2021 Aug 31;12:702558. doi: 10.3389/fendo.2021.702558 (PMC8438422; doi:10.3389/fendo.2021.702558)
Supplement: Supplementary file 1 [file DataSheet_1.docx]

Supplementary Material

# Supplementary Appendix 1: search strategy

Pubmed

#1 Progestins[MeSH]

#2 Progesterone[MeSH]

#3 Desogestrel[MeSH]

#4 Dydrogesterone[MeSH]

#5 "progestin-primed ovarian stimulation"[Title/Abstract]

#6 progest*[Title/Abstract]

#7 "Gestagen"[Title/Abstract]

#8 "dydrogesterone*"[Title/Abstract]

#9 "duphaston"[Title/Abstract]

#10 "gestrinone*"[Title/Abstract]

#11 utrogestan[Title/Abstract]

#12 medroxyprogesterone*[Title/Abstract]

#13 medrogestone[Title/Abstract]

#14 depoprovera[Title/Abstract]

#15 "depot medroxyprogesterone"[Title/Abstract]

#16 #1 OR #2 OR #3 OR #4 OR #5 OR #6 OR #7 OR #8 OR #9 OR #10 OR #11 OR #12 OR #13 OR #14 OR #15

#17 Ovulation Induction[MeSH]

#18 Superovulation [MeSH]

#19 Fertilization in Vitro [MeSH]

#20 sperm injections, intracytoplasmic [MeSH]

#21 Reproductive Techniques [MeSH]

#22 "ovarian stimulation"[Title/Abstract]

#23 "assisted reproduction"[Title/Abstract]

#24 "vitro fertili*"[Title/Abstract]

#25 "ivf"[Title/Abstract]

#26 "icsi"[Title/Abstract]

#27 "COH"[Title/Abstract]

#28 "ART"[Title/Abstract]

#29 "ovulation"[Title/Abstract]

#30 "intracytoplasmic sperm injections" [Title/Abstract]

#31 "Subfertility" [Title/Abstract]

#32 "Infertility"[Title/Abstract]

#33 #17 OR #18 #OR #19 OR #20 OR #21 OR #22 OR #23 OR #24 OR #25 OR #26 OR #27 OR #28 OR #29 OR #30 OR #31 OR #32

#34 **"Cetrotide"[Title/Abstract]**

**#35 "cetrorelix*"[Title/Abstract]**

**#36 "Premature LH surge"[Title/Abstract]**

**#37 "LHRH antagonist"[Title/Abstract]**

**#38 "antagonist"[Title/Abstract]**

**#39 "agonist"[Title/Abstract]**

**#40 "GnRH antagonist"[Title/Abstract]**

**#41 "gonadotropins*"[Title/Abstract]**

**#42 "Menotropins"[Title/Abstract]**

**#43 "GnRH agonist"[Title/Abstract]**

**#44 "GnRHa*"[ Title/Abstract]**

**#45 "premature ovulation"[ Title/Abstract])**

#46 #34 OR #35 OR #36 OR #37 OR #38 OR #39 OR #40 OR #41 OR #42 OR #43 OR #44 OR #45

#47 #16 AND #33 AND #46

Cochrane:

#1 MeSH descriptor:[Progestins] explode all tree

#2 MeSH descriptor:[Progesterone] explode all tree

#3 MeSH descriptor:[Desogestrel] explode all tree

#4 MeSH descriptor:[Dydrogesterone] explode all tree

#5 ("primed ovarian stimulation"):ti,ab,kw

#6 (progest*):ti,ab,kw

#7 (Gestagen):ti,ab,kw

#8 (dydrogesterone*):ti,ab,kw

#9 (duphaston):ti,ab,kw

#10 (gestrinone*):ti,ab,kw

#11 (utrogestan):ti,ab,kw

#12 (medroxyprogesterone*):ti,ab,kw

#13 (medrogestone):ti,ab,kw

#14 (depoprovera):ti,ab,kw

#15 ("depot medroxyprogesterone"):ti,ab,kw

#16 #1 OR #2 OR #3 OR #4 OR #5 OR #6 OR #7 OR #8 OR #9 OR #10 OR #11 OR #12 OR #13 OR #14 OR #15

#17 MeSH descriptor:[Reproductive Techniques] explode all tree

#18 ("ovarian stimulation"):ti,ab,kw

#19 ("assisted reproduction"):ti,ab,kw

#20 ("in vitro fertilization"):ti,ab,kw

#21 (ivf):ti,ab,kw

#22 (icsi):ti,ab,kw

#23 (COH):ti,ab,kw

#24 (ART):ti,ab,kw

#25 (ovulation):ti,ab,kw

#26 ("intracytoplasmic sperm injections" ):ti,ab,kw

#27 (Subfertility):ti,ab,kw

#28 (Infertility):ti,ab,kw

#29 #17 OR #18 #OR #19 OR #20 OR #21 OR #22 OR #23 OR #24 OR #25 OR #26 OR #27 OR #28

#30 **(Cetrotide**):ti,ab,kw

**#31 (cetrorelix***):ti,ab,kw

**#32 (‘Premature LH surge"**):ti,ab,kw

**#33 (“LHRH antagonist"**):ti,ab,kw

**#34 (antagonist**):ti,ab,kw

**#35 (agonist**):ti,ab,kw

**#36 (“GnRH antagonist"**):ti,ab,kw

**#37 (gonadotropins***):ti,ab,kw

**#38 (Menotropins**):ti,ab,kw

**#39 (“GnRH agonist"[Title/Abstract]**

**#40 (GnRHa***):ti,ab,kw

**#41 (“premature ovulation"**):ti,ab,kw

#42 #33 OR #34 OR #35 OR #36 OR #37 OR #38 OR #39 OR #40 OR #41

#43 #16 AND #29 AND #42

EMBASE

#1 ('gestagen'/exp) AND [2010-2020]/py

#2 ('primed ovarian stimulation':ab,ti OR 'progestin*':ab,ti OR 'progesteron*':ab,ti OR 'progestogen*':ab,ti OR 'progesta*':ab,ti OR 'gestagen':ab,ti OR 'dydrogesterone*':ab,ti OR 'duphaston':ab,ti OR 'gestrinone*':ab,ti OR 'utrogestan':ab,ti OR 'medroxyprogesterone*':ab,ti OR 'medrogestone':ab,ti OR 'depoprovera':ab,ti OR 'depot medroxyprogesterone':ab,ti) AND [2015-2020]/py

#3 #1 OR #2

#4 ('ovulation induction'/exp OR 'superovulation'/exp OR 'in vitro fertilization'/exp OR 'reproductive procedure'/exp) AND [2015-2020]/py

#5 ('ovarian stimulation':ab,ti OR 'assisted reproduction':ab,ti OR 'vitro fertili*':ab,ti OR 'ivf':ab,ti OR 'icsi':ab,ti OR 'coh':ab,ti OR 'art':ab,ti OR 'ovulation':ab,ti OR 'intracytoplasmic sperm injections':ab,ti OR 'subfertility':ab,ti OR 'infertility':ab,ti) AND [2015-2020]/py

#6 #4 OR #5

#7 ('cetrotide':ab,ti OR 'cetrorelix*':ab,ti OR 'premature lh surge':ab,ti OR 'lhrh antagonist':ab,ti OR 'antagonist':ab,ti OR 'agonist':ab,ti OR 'gnrh antagonist':ab,ti OR 'gonadotropins*':ab,ti OR 'menotropins':ab,ti OR 'gnrh agonist':ab,ti OR 'gnrha*':ab,ti) AND [2015-2020]/py

#8 # AND #6 AND #7

Web of science

#1 TS=(gestagen) OR TS=(progest*) OR TS=(desogestrel) OR TS=(dydrogesterone*) OR TS=(medroxyprogesterone*) OR TS=(medrogestone) OR TS=(depoprovera) OR TS=("depot medroxyprogesterone") OR TS=('primed ovarian stimulation') OR TS=(gestrinone*) OR TS=(utrogestan)

#2 TS=(Ovulation) OR TS=(Superovulation) OR TS=('in vitro fertilization') OR TS=("Reproductive Techniques") OR TS=("ovarian stimulation") OR TS=("assisted reproduction") OR TS=("vitro fertili*") OR TS=(Subfertility) OR TS=("Infertility") OR TS=(IVF) OR TS=(ICSI) OR TS=(ART) OR TS=(COH)

#3 TS=("Cetrotide") OR TS=("cetrorelix*") OR TS=("Premature LH surge") OR TS=("LHRH antagonist") OR TS=("antagonist") OR TS=("agonist") OR TS=("GnRH antagonist") OR TS=("gonadotropins*") OR TS=("Menotropins") OR TS=("GnRH agonist") OR TS=("GnRHa*") OR TS=("premature ovulation")

#4 #1 AND #2 AND #3
