# Supplementary material for: Progestin-Primed Ovarian Stimulation Protocol for Patients in Assisted Reproductive Technology: A Meta-Analysis of Randomized Controlled Trials
Source: Front Endocrinol (Lausanne). 2021 Aug 31;12:702558. doi: 10.3389/fendo.2021.702558 (PMC8438422; doi:10.3389/fendo.2021.702558)
Supplement: Supplementary file 2 [file DataSheet_2.docx]

Supplementary Material

# Supplementary Appendix 2

**Figure 1** Forest plot of studies of Gn duration

**Figure 2** Forest plot of studies of Gn dose

**Figure 3** Forest plot of studies of LH on trigger day

**Figure 4** Forest plot of studies of oocytes retrieved

**Figure 5** Forest plot of studies of MII oocytes

**Figure 6** Forest plot of studies of viable embryos

**Figure 7** Forest plot of studies of miscarriage rate
