# Supplementary material for: Progestin-Primed Ovarian Stimulation Protocol for Patients in Assisted Reproductive Technology: A Meta-Analysis of Randomized Controlled Trials
Source: Front Endocrinol (Lausanne). 2021 Aug 31;12:702558. doi: 10.3389/fendo.2021.702558 (PMC8438422; doi:10.3389/fendo.2021.702558)
Supplement: Supplementary file 3 [file DataSheet_3.docx]

Supplementary Material

# Supplementary Appendix 3

**Figure 1** Forest plot of studies of premature LH surge

**Figure 2** Forest plot of studies of clinical pregnancy rate per woman

**Figure 3** Forest plot of studies of live birth or ongoing pregnant rate per woman

**Figure 4** Forest plot of studies of OHSS
